# Gold exploration in the Gabal Abu Karahish area, Central Eastern Desert, Egypt: an integrated geological perspective

**DOI:** 10.1038/s41598-025-04496-1

**Published:** 2025-06-05

**Authors:** Mahmoud Abd El-Rahman Hegab, Salem Mohamed Salem, Nehal Mohamed Soliman, Sobhi Mahmoud Ghoneim, Kareem Hamed Abd El Wahid, Hala Fouad Ali, Mohamed Anwar Ahmed

**Affiliations:** National Authority for Remote Sensing and Space Sciences, Cairo, Egypt

**Keywords:** ASTER, Landsat-9, Gold, Alteration zones, Abu Karahish area, Geology, Economic geology

## Abstract

This study aims to explore the presence and distribution of gold deposits in the Gabal Abu Karahish area by identifying hydrothermal alteration zones associated with favorable geological settings. The objective is to assess gold potential through an integrated remote sensing and geochemical approach. Multispectral satellite data from ASTER and Landsat-9, combined with radiometric data and field geology, were utilized to delineate alteration zones indicative of mineralization. ASTER band ratios (7/6, 4/6, and 9/8) and Landsat-9 false color composites were processed to enhance lithological discrimination and detect hydrothermal alterations. Automated lineament extraction was also performed to evaluate structural controls on mineralization. Several alteration zones of argillic, phyllic, and propylitic types were identified and are spatially associated with alteration minerals such as chlorite, calcite, kaolinite, sericite, and iron oxides. Scanning electron microscopy (SEM) analysis of ten representative samples from alteration zones and quartz veins in metavolcanic and ultramafic rocks confirmed the presence of gold in all samples, with concentrations ranging from 0.23 to 0.83 g per 50 g of rock powder. These findings highlight key zones for further gold exploration. Geologically, the area is composed of calc-alkaline metavolcanic rocks, Dokhan volcanic rocks, serpentinites, talc carbonates, hornblende gabbros, tonalite, granodiorite, and younger granite intrusions. The lithological diversity and structural features, including listwanite ridges and overthrust contacts, further support the area’s mineral potential.

## Introduction

Gold mineralization is commonly associated with hydrothermal alteration zones formed by fluid circulation along structural features such as faults, shear zones, and fractures. These zones often contain a suite of alteration minerals, such as chlorite, sericite, kaolinite, and iron oxides, which serve as indicators of ore-forming processes^1–4^. The integration of remote sensing, geological, and geochemical tools has proven to be an effective strategy for identifying these zones, particularly in arid and inaccessible terrains.

In the Central Eastern Desert of Egypt, the geological setting is characterized by Neoproterozoic basement rocks, including metavolcanics, serpentinites, and intrusive granitoids, all of which are highly deformed and structurally complex. This tectonically active environment is favorable for hydrothermal fluid migration and gold mineralization^5–7^. Hydrothermal fluids, driven either by metamorphism or by the cooling of early Cambrian subduction-related calc-alkaline magmatism, have the potential to generate a variety of mineral deposits. These mineral-bearing solutions typically migrate along rock lineaments and fractures, resulting in alteration zones enriched in economically valuable minerals^2,4,5,7^.

Previous regional studies in the Eastern Desert have demonstrated the effectiveness of remote sensing tools, particularly ASTER, in mapping alteration zones associated with gold deposits^8–10^. However, detailed studies specifically focusing on the Gabal Abu Karahish–Gabal Semna area using an integrated approach involving ASTER, Landsat-9, and SEM analysis remain limited.

In this context, ASTER’s six SWIR bands allow for effective differentiation of altered minerals, while Landsat-9 imagery provides complementary data for structural and lithological analysis^2,8,10–12^. The combination of remote sensing, fieldwork, and geochemical analysis enhances the ability to detect and characterize hydrothermal alteration zones with potential gold mineralization^13–18^.

Therefore, the objective of this study is to identify and characterize hydrothermal alteration zones associated with gold mineralization in the Gabal Abu Karahish–Gabal Semna area using integrated remote sensing (ASTER and Landsat-9), field geology, and SEM analysis. The study also aims to evaluate the relationship between these alteration zones, host lithologies, and structural features to guide future exploration efforts.

## Study area and geology

**Fig. 1:**
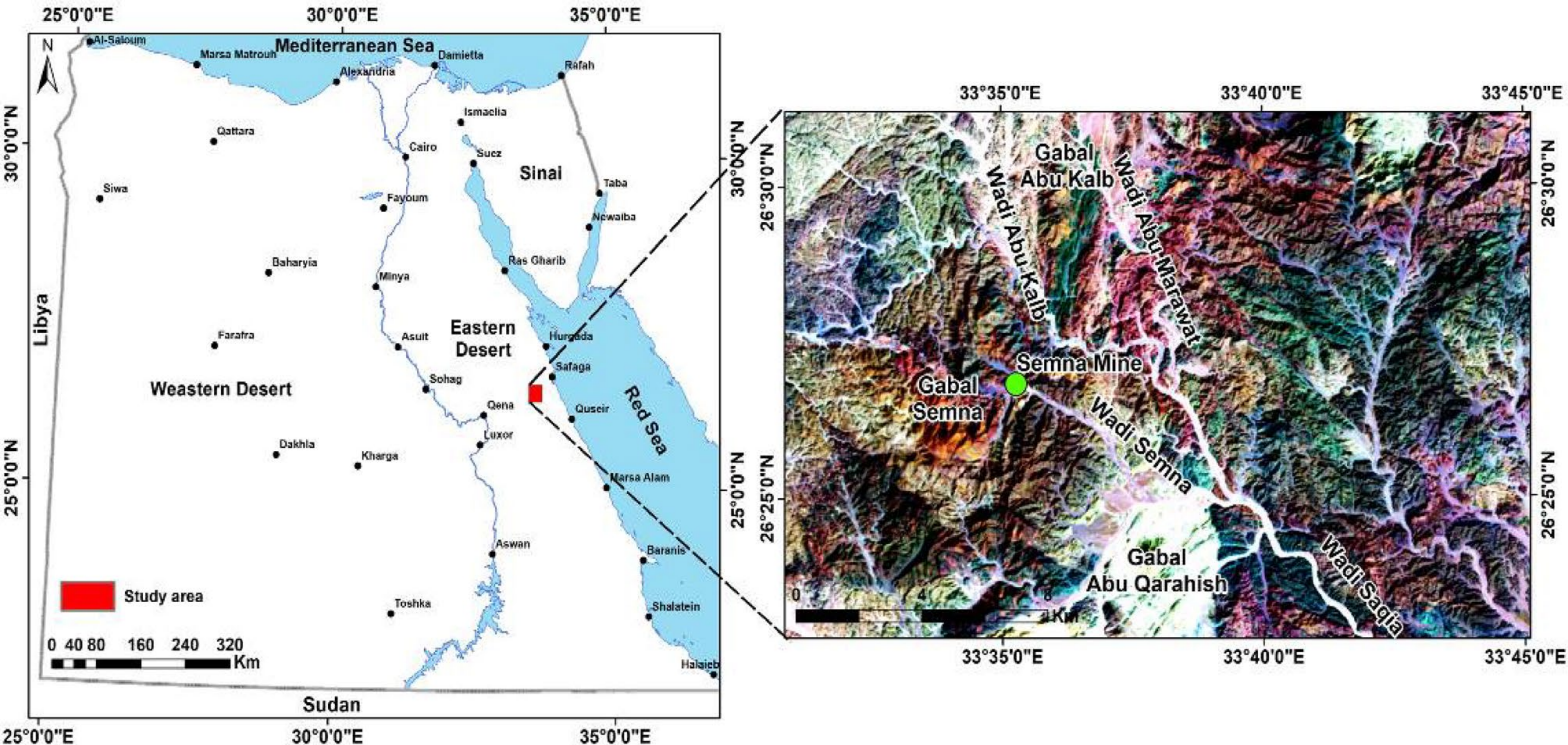
Location map of the Gabal Abu Karahish area. (By ArcGIS v.10.5. https://www.esri.com/en-us/arcgis/products/arcgis-desktop/overview/).

**Fig. 2:**
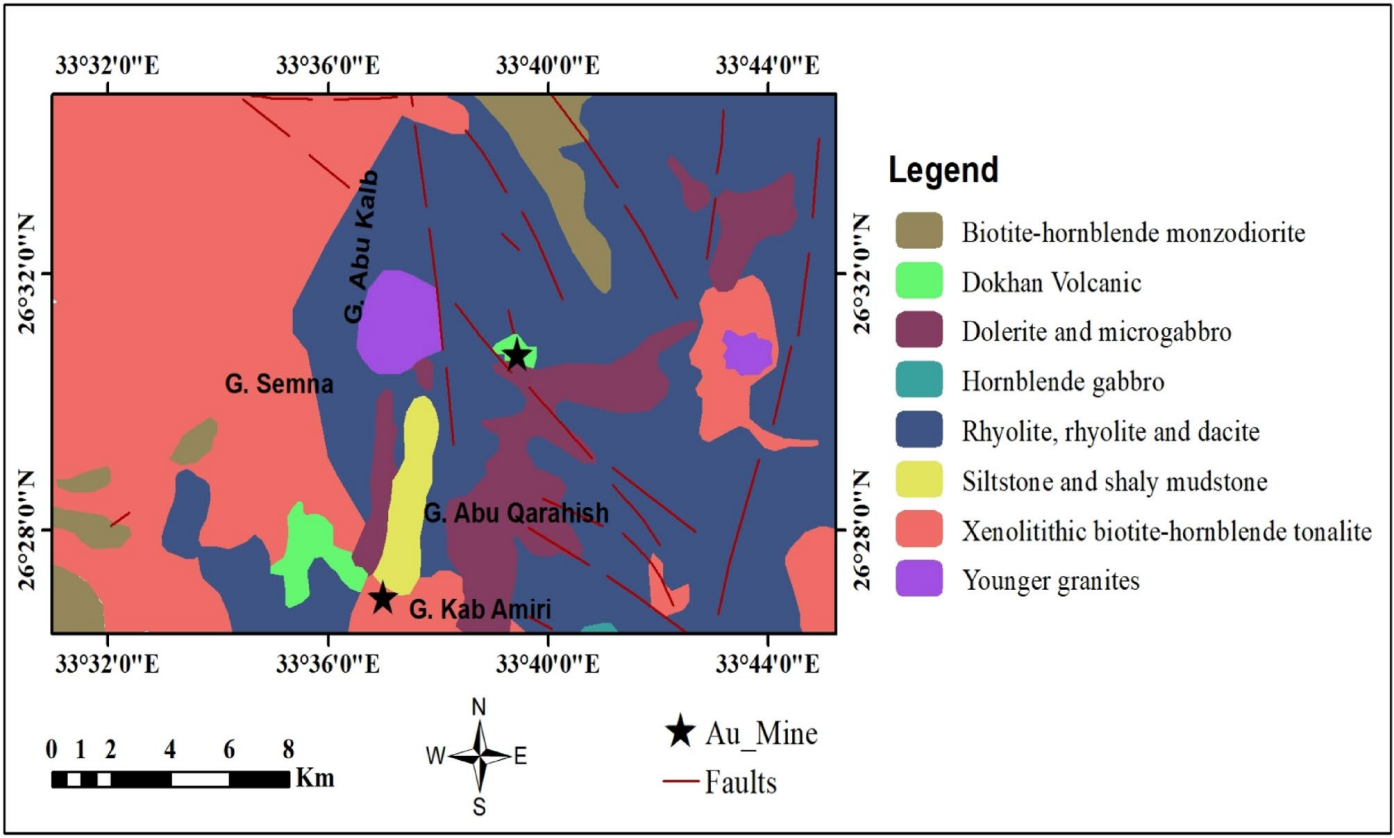
Geological map of the study area (modified after EGSMA^19^). (By ArcGIS v.10.5. https://www.esri.com/en-us/arcgis/products/arcgis-desktop/overview/).

The study area is located in the Central Eastern Desert, south of the Safaga–Qena road and the Abu Marawat mine, directly east of Wadi Saqia (Fig. 1). The rock units exposed in the Gabal Abu Karahish area are listed in chronological order, with the youngest formations on top (Fig. 2). A sequence of banded iron formations (BIFs) is present and hosted within the metavolcanic rocks in the northern part of the Gabal Abu Karahish area, where these metavolcanic rocks are overlain by serpentinite masses and talc carbonates^19^. The Dokhan volcanics primarily consist of massive and laminated porphyritic rhyolite and rhyodacite, with andesite being less common. A few hornblende gabbros and small tonalite–granodiorite bodies intrude the metavolcanic and ultramafic rocks. Additionally, younger granites from Gabal Abu Karahish, Gabal Wae’ra, and Gabal Kab Amiri have intruded into the metavolcanic rocks and may be associated with mineral-bearing hydrothermal solutions and the development of alteration zones. The study area is predominantly composed of older metavolcanic rocks. The subaerial Dokhan volcanic rocks, which include quartz-feldspar porphyry and andesite porphyry, are interlayered with siltstone, mudstone, and polymictic conglomerates typical of molasse-type Hammamat sediments^5,7,20^.

## Materials and methods

**Fig. 3:**
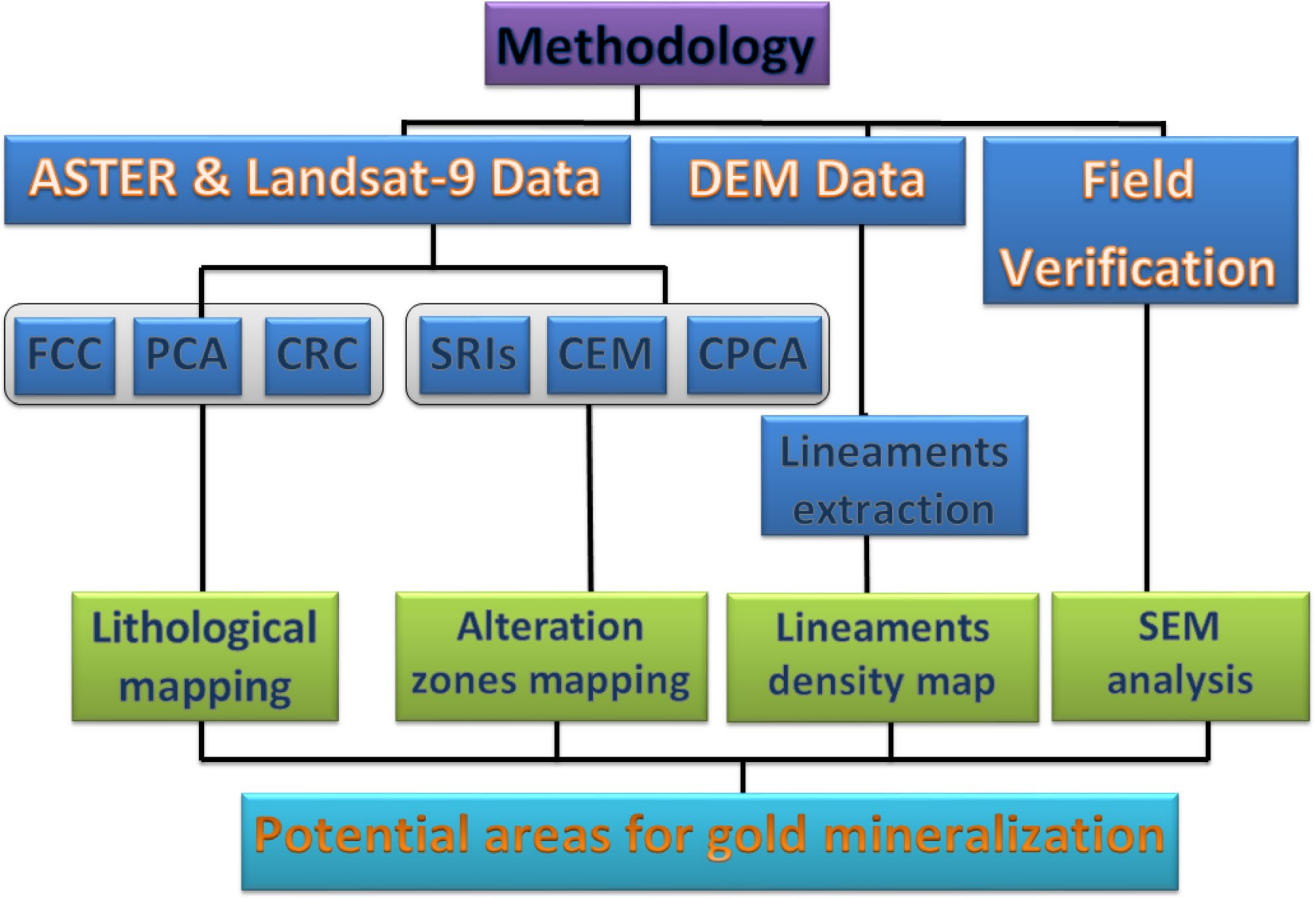
The methodology flowchart.

The methodological framework of this study was constructed by integrating remote sensing (Landsat-9 (OLI) and ASTER L1T) data obtained from the (https://earthexplorer.usgs.gov) website. Landsat-9, the most recent satellite in the Landsat lineage, introduces cutting-edge capabilities suited for a variety of remote sensing purposes. It features an extensive set of spectral bands, including Visible (VIS), Near-Infrared (NIR), Shortwave Infrared (SWIR), Panchromatic (PAN), Thermal Infrared (TIR), and a Cirrus band, allowing it to observe multiple dimensions of the Earth’s surface. Resolution varies by band: 30 m for VIS, NIR, and SWIR; 15 m for PAN; and 100 m for TIR bands 10 and 11. Meanwhile, the ASTER sensor delivers broad spectral coverage across Visible Near Infrared (VNIR), SWIR, and Thermal Infrared (TIR) domains. Its VNIR includes three bands: band 1 (0.52 to 0.60 μm), band 2 (0.63 to 0.69 μm), and band 3 N (0.78 to 0.86 μm), offering detailed surface reflectance data. In the SWIR range, ASTER provides six bands (4 to 9) from 1.60 to 2.430 μm, and in TIR, it includes five bands (10 to 14) spanning 8.125 to 11.65 μm. ASTER’s spatial resolution differs across these regions: 15 m for VNIR, 30 m for SWIR, and 90 m for TIR. This study employed an integrated remote sensing approach to map lithological units, extract lineaments, and identify alteration zones in the target area, utilizing Landsat-9 and ASTER satellite data processed with advanced software tools such as ENVI and ArcGIS v.10.5, as illustrated in Fig. 3.

For lithological mapping using Landsat-9, false color composite (FCC) images were generated by selecting optimal band triplets based on correlation coefficients (CCs) and the Optimum Index Factor (OIF). The CC method assessed inter-band relationships, identifying low-correlation triplets, while the OIF ranked combinations that maximized variance and minimized redundancy. RGB combinations were visually selected to enhance rock discrimination. Principal Component Analysis (PCA) was applied to VNIR-SWIR bands, capturing 99.9% of the data variance, with selected components used to highlight rock units and structures. Color ratio composites (CRC) further distinguish lithological units and alteration zones. Automatic lineament extraction involved generating shaded relief images from ALOS PALSAR DEM data in ArcGIS, producing lineament and density maps based on the number of lineaments per km².

For alteration mapping with ASTER, spectral ratio indices (SRIs) like OHI, KLI, CLI, and b2/b1 identified OH-bearing minerals, kaolinite, and iron oxides. The Constrained Energy Minimization (CEM) technique mapped dominant altered minerals (e.g., kaolinite, illite, iron oxides) using VNIR-SWIR reflectance data, while Crosta PCA (CPCA) analyzed specific bands (e.g., 4-6-7-9 for kaolinite) to delineate alteration minerals like alunite and montmorillonite. Finally, spatial correlation between lineament density and alteration maps highlighted mineral exploration targets, such as the Semna and Abu Qarahish zones, integrating all datasets for a comprehensive geological assessment.

The results of the image processing were proven by fieldwork, where geological explanations and (Global Position System) GPS validation points were gathered to check the lithology and alteration zones provided by remote sensing. Several altered rock samples were composed of various rock units for scanning electron microscopy (SEM) and field spectral measurements. These tools were integrated and helped in lithological discrimination and mapping of alteration zones as target areas for gold exploration^2,11,21–24^. The Landsat-9 and ASTER satellite data were preprocessed to retrieve surface reflectance values for each pixel and to correct errors caused by atmospheric interactions. Spatial resolution merging and area of interest steps were used. Then, numerous image processing approaches were applied to the Landsat-9 satellite data to identify the various lithological units to display the spatial distribution of the alteration zones.

## Results

### Lithological mapping using Landsat-9 data

#### False color composite (FCC)

The optimal band triplets for false color composite images were selected using two methods: correlation coefficients (CCs) and the optimum index factor (OIF)^8,11,12,14^. The correlation coefficient method is a statistical approach used to assess the strength of the linear relationship between variables^12,15,16^. A smaller correlation coefficient signifies a higher level of variance in the data^2,25^. By using ENVI image processing software, the correlation coefficient between every two bands was obtained for the seven VNIR-SWIR reflective bands and the correlation matrix (Table 1). The correlation coefficient results revealed a positive correlation between all seven VNIR-SWIR bands. The correlation coefficients for the ten band triplets are presented and ranked in Table 2.

**Table 1:** The correlation coefficients of Landsat-9 bands.

Bands	Band1	Band2	Band3	Band4	Band5	Band6	Band7
Band1	1						
Band2	0.998121	1					
Band3	0.989656	0.992927	1				
Band4	0.968145	0.973071	0.989795	1			
Band5	0.939839	0.945918	0.96826	0.992534	1		
Band6	0.906028	0.911217	0.936791	0.956796	0.964928	1	
Band7	0.896672	0.900558	0.923468	0.937092	0.940756	0.980317	1

**Table 2:** The top ten band triplets for Landsat-9 data in the study area were ranked using the correlation coefficient method.

Band Triplet	Correlation coefficients	Rank
157	2.777267	1
167	2.783017	2
257	2.787232	3
267	2.792092	4
127	2.795351	5
147	2.801909	6
137	2.809796	7
247	2.810721	8
156	2.810795	9
126	2.815366	10

The optimum index factor (OIF) is a statistical calculation used to evaluate all possible three-band combinations, such as RGB^26,27^. The OIF values are computed to identify the most favorable band combinations^28^, and the band triplets are ranked based on the information contained in each combination^29^. The potential for RGB visualization is determined by the total variance and correlation between different bands^28,29^. Triplets with higher OIF values were selected for better lithological discrimination, as they include bands with maximum variance and minimal redundancy^27–29^. The top ten band triplet combinations are listed in Table 3.

**Table 3:** Ranking of the Landsat-9 data for the study area using the optimum index factor (OIF).

Band Triplet	OIF	Rank
567	0.068248	1
467	0.065993	2
456	0.063795	3
367	0.062326	4
457	0.062199	5
356	0.060376	6
267	0.058863	7
357	0.05857	8
167	0.057951	9
567	0.068248	10

**Fig. 4:**
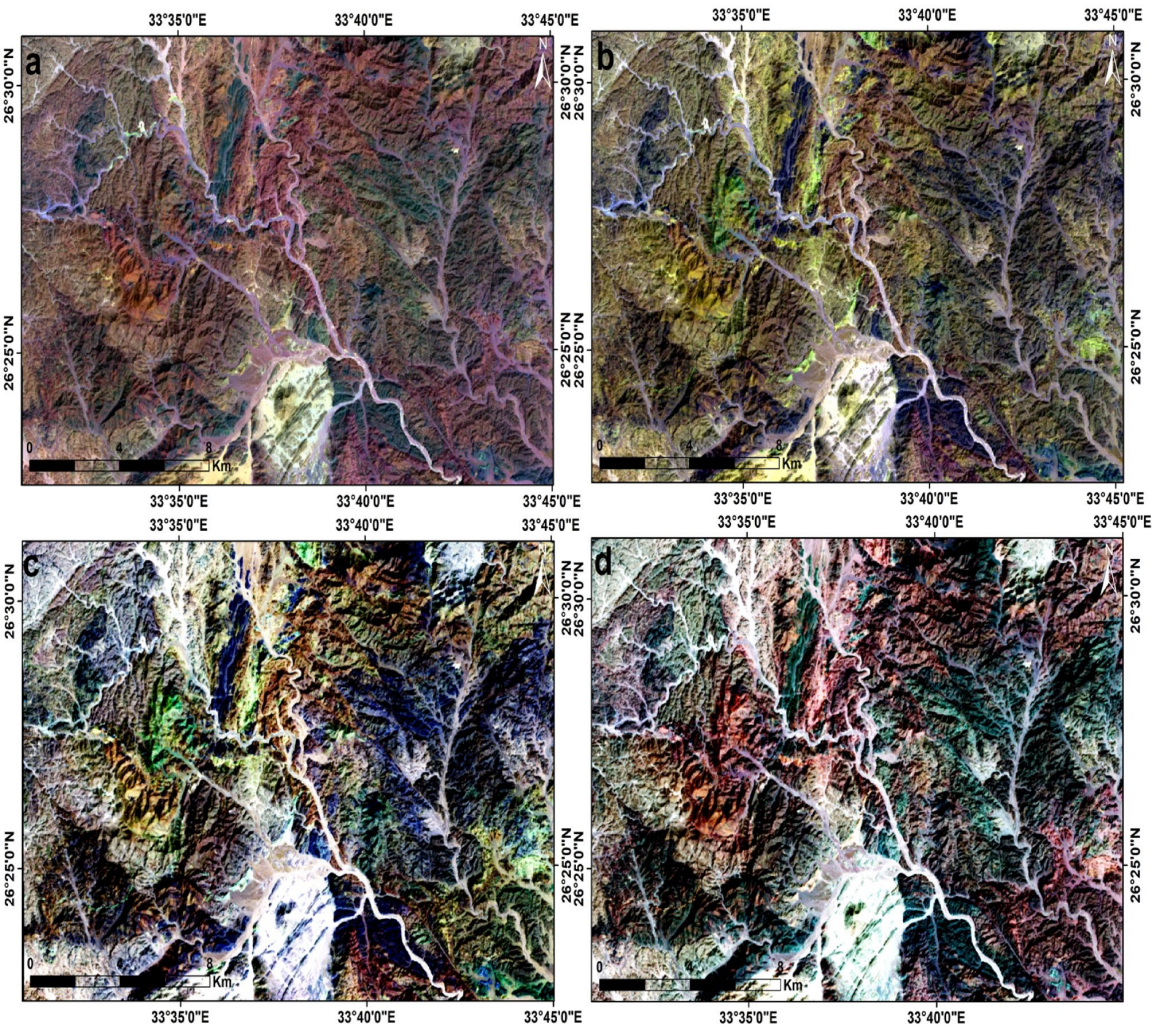
Landsat-9 FCC images (**a**) band7, band5, band1 (**b**) band7, band6, band1 (**c**) band7, band6, band5, and (**d**) band6, band5, band4 in RGB, respectively. (By ENVI https://www.l3harrisgeospatial.com/Software-Technology/ENVI).

Through visual inspection of the selected best band triplet possibilities obtained from both the CC and OIF methods, RGB images 7, 5, 1, 7, 6, 2, 7, 2, 1 and 7, 4, 1 in RGB were selected for better lithological discrimination of the exposed rocks in the area (Fig. 4, a, b,c, d).

#### Principal component analysis (PCA)

The advantage of PCA is that in multispectral data, the majority of the variance is usually concentrated in the first three or four principal component images^11,13,30^. Noise tends to accumulate in the less correlated principal components. The first principal component (PC) image captures the most variance and has the lowest correlation, while the last PC image contains the least variance and highest correlation, making the lower-order principal components less useful (e.g., the last principal component image) appear noisy and one cannot identify the features in it clearly^2,12,31^.

**Fig. 5:**
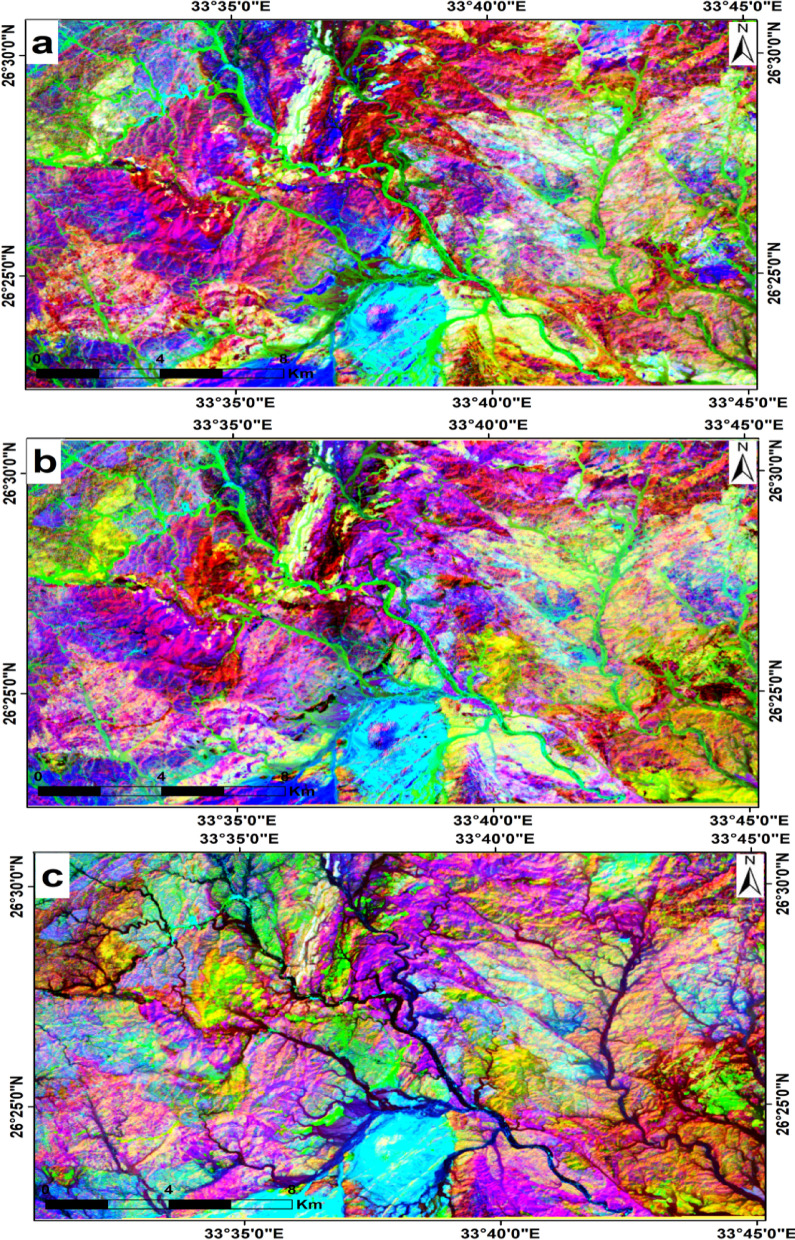
Landsat-9 false color composites (FCCs) created using different combinations of principal components (PCs) displayed in RGB: (**a**) PC3–PC2–PC1, (**b**) PC4–PC2–PC1, and (**c**) PC4–PC3–PC1. (By ENVI https://www.l3harrisgeospatial.com/Software-Technology/ENVI).

The principal components were computed from the VNIR and SWIR data bands in the present study. According to the analysis of the eigenvector matrix (Table 4), PC1, 2, 3, and 4 contained almost 99.9% of the information in the data, while the other PC bands contained less than 0.1%. Hence PC3, PC2, and PC1; PC4 and PC2 and PC1 were used, so they contained the most informative data with the highest variance. These data clearly show the relationships between the different rock units and illustrate the general structural features flanking these rocks (Fig. 5a, b, c).

**Table 4:** Eigenvector matrix and loadings of principal component analysis (PCA) on Landsat-9 images.

	PC 1	PC 2	PC 3	PC 4	PC 5	PC 6	PC 7
Band 1	-0.94170	-0.32103	0.09485	-0.01849	-0.02782	-0.00108	-0.00476
Band 2	0.24145	-0.84451	-0.45793	-0.10892	0.08302	0.00647	0.00090
Band 3	0.20250	-0.34696	0.84898	-0.26582	0.21518	0.00409	0.02947
Band 4	-0.05334	0.17872	-0.16485	-0.95084	-0.18250	-0.01088	-0.02274
Band 5	-0.10342	0.17392	-0.17947	-0.11301	0.91483	0.18942	0.20321
Band 6	0.01662	-0.03269	0.02497	0.01515	-0.27058	0.49285	0.82564
Band 7	-0.00873	0.00984	-0.02296	-0.00632	0.05106	-0.84914	0.52499
Eigenvalues	**0.01824**	**0.00046**	**0.00015**	**0.00006**	**0.00001**	**0.00000**	**0.00000**
	**0.96401**	**0.02442**	**0.00766**	**0.00328**	**0.00048**	**0.00011**	**0.00005**
%	**96.40**	**2.44**	**0.77**	**0.33**	**0.05**	**0.01**	**0.01**

#### Color ratio composites (CRC)

This method effectively distinguishes between rock types by highlighting the primary differences in the visible, near-infrared, and shortwave-infrared spectral regions, which are evident along the slopes of the reflectivity curves. The rationing process mitigates first-order brightness effects induced by topographic slopes and accentuates subtle color variations between materials^32^.

**Fig. 6:**
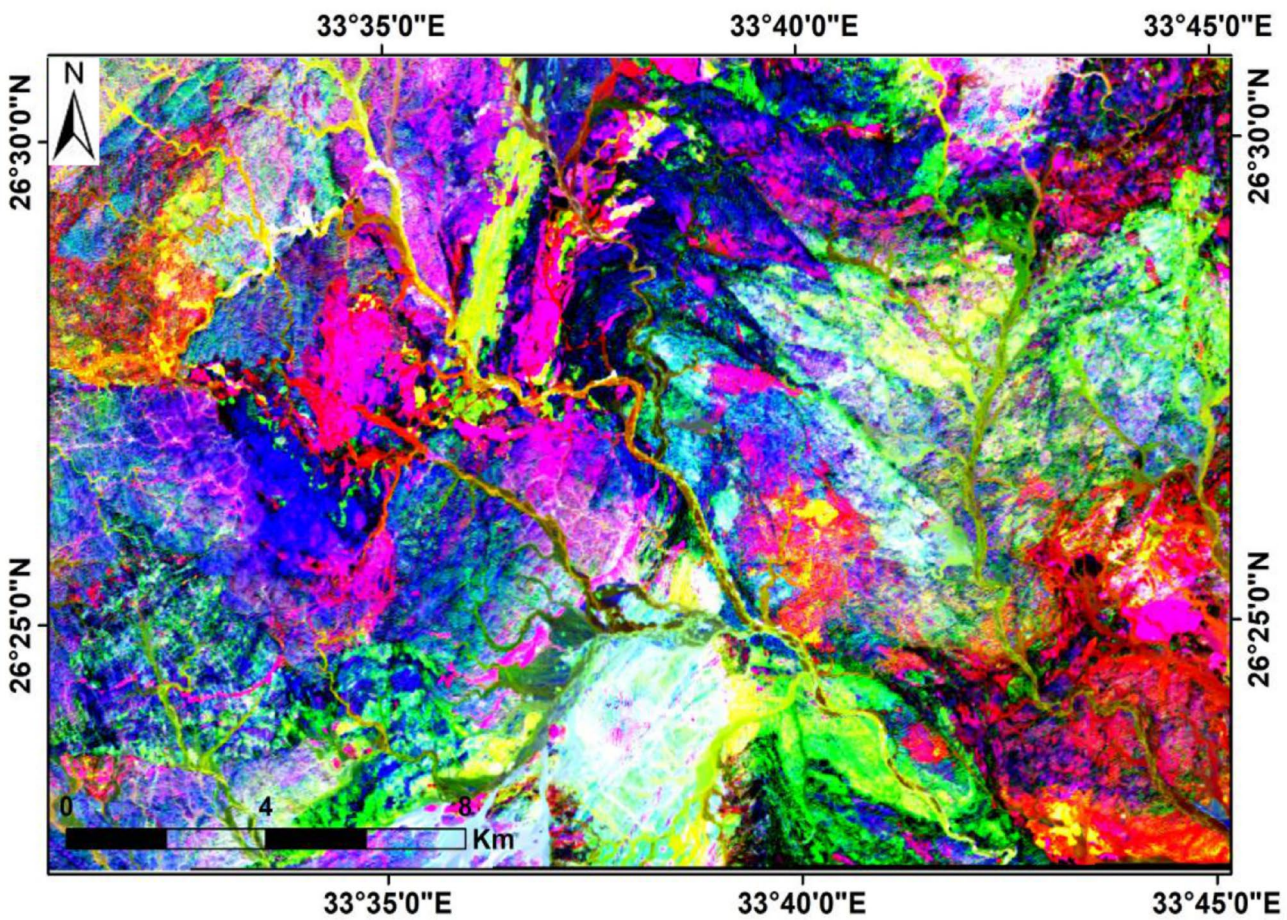
Landsat-9 band ratio composite using 6/7 (Red), 5/6 (Green), and 4/2 (Blue) in RGB, modified after Abrams et al. (1983). (By ENVI https://www.l3harrisgeospatial.com/Software-Technology/ENVI).

**Fig. 7:**
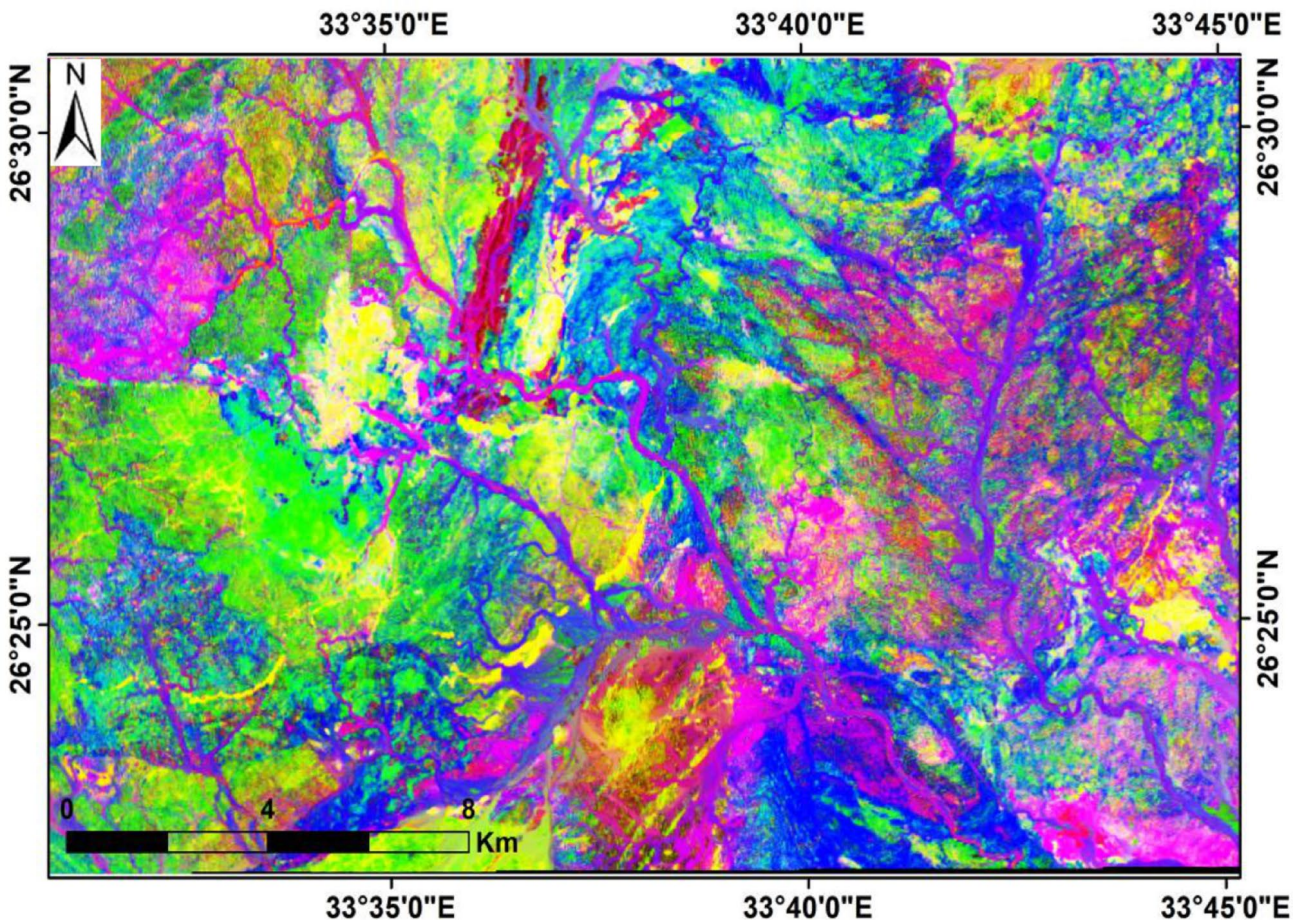
Landsat-9 band ratio composite using RGB: 6/7 (Red), 5/2 (Green), and (4/5) × (6/5) (Blue), modified after Sultan et al. (1986). (By ENVI https://www.l3harrisgeospatial.com/Software-Technology/ENVI).

Ratio images are generated by dividing the digital number, radiance, or reflectance values of one band by those of another band for each pixel. The resulting ratios are subsequently normalized and visualized as a new ratio image^33,34^. In the present study, the composite ratios (6/7, 5/6 and 4/2) in RGB were verified to be the optimum mixtures for lithological discrimination and differentiation of the alteration zones from their host rocks (Fig. 6). Moreover, various ratios (6/7, 5/2 and 4/5*6/5) were also used for alteration mapping, which mainly appeared yellow to yellowish green colors (Fig. 7).

### Automatic lineament extraction

Extracting lineaments from satellite remotely sensed data to map zones of weakness, such as faults, joints, cracks, fissures or other linear features is becoming a common automated process. The importance of mapping linear features is that they represent channels and paths for mineral-bearing hydrothermal fluids. Hydrothermal chemical activity within the surrounding country rocks led to the development of haloes in alteration zones within and around the lineament zones. The types and extensions of these alterations depend on the composition and intensity of such solutions. The lineaments represent areas of deformation and higher secondary porosity and are significant channels for fluid migration.

Several researchers have explored lineament extraction using various remote sensing techniques and datasets. Some studies focused on selecting the optimal Landsat ETM + bands for automatic lineament extraction and analyzing the correlation between extracted lineament trends and uranium occurrences in granitic rocks^35^. Others utilized digital Landsat ETM + imagery and processed it with Geo-analyst PCI software to extract lineaments in the Gabal Gharib-Dara area^36^. Additionally, researchers employed SPOT imagery within the Geomatica PCI package to identify lineaments in the Petra-Wadi Al Lahyana region of Jordan^37^. Furthermore, lineaments were derived from multiple hillshade images generated with varying illumination azimuths (0°, 45°, 90°, 135°, 180°, 225°, 270°, and 315°) using ALOS PALSAR DEM data, processed in ArcMap v.10.5^22^.

**Fig. 8:**
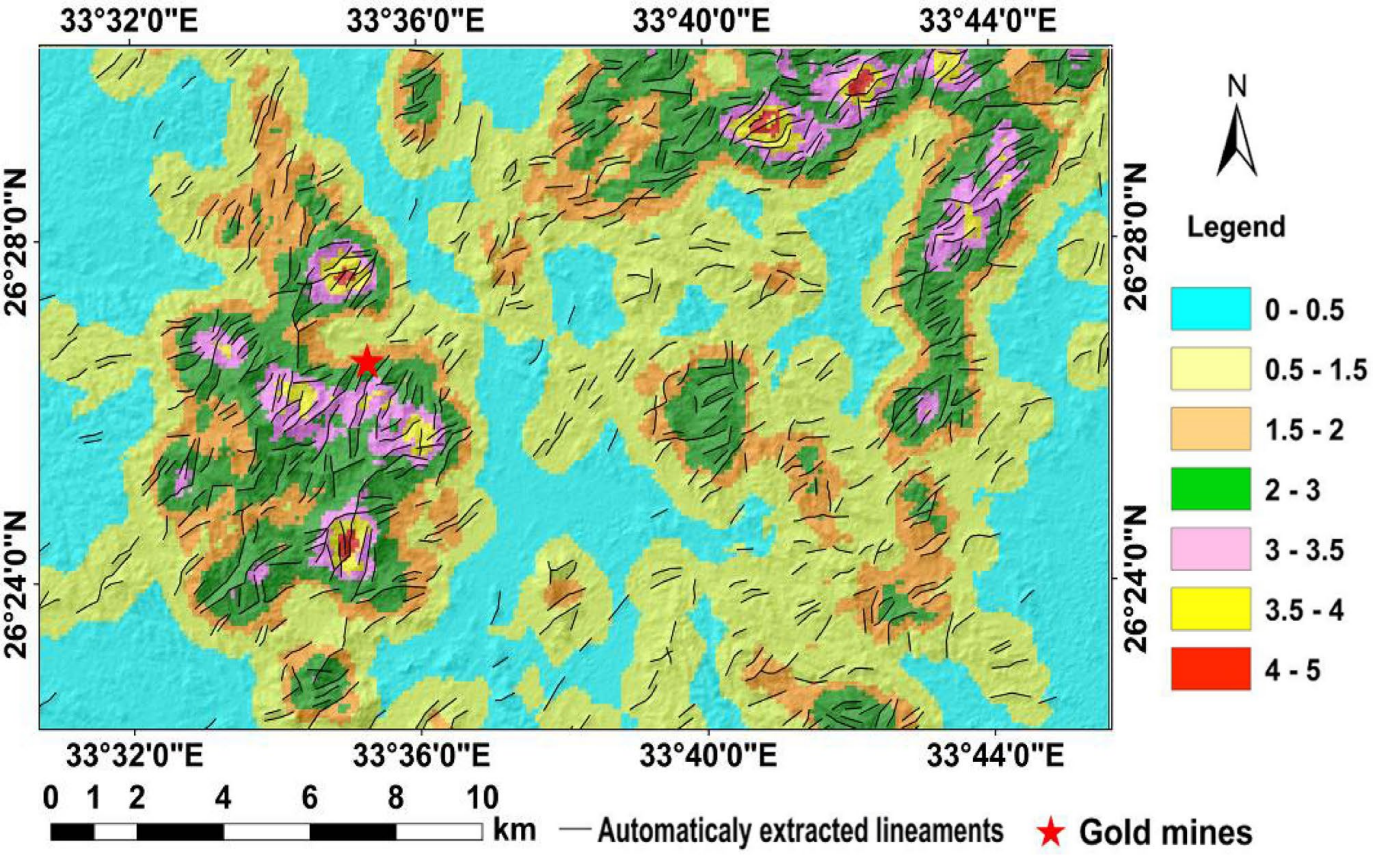
Lineament density map of the study area, classified into seven ranges (0–5 number/km²). Black lines represent automatically extracted lineaments, and red stars mark known gold mines. High-density zones (pink to red) show strong spatial correlation with structural trends and gold mineralization. (By ArcGIS v.10.5. https://www.esri.com/en-us/arcgis/products/arcgis-desktop/overview/).

Lineaments were extracted from shaded relief images and correlated with alteration data to identify areas with high potential for mineral exploration^38^. Lineament density was calculated using ArcGIS v.10.5, based on the number of lineaments per unit area (number/km²). This is one of the two common methods for calculating lineament density; the other involves summing the total length of lineaments per unit area (km/km²). The resulting lineament density map (Fig. 8) illustrates the frequency and concentration of lineaments across the study area. High-density zones often coincide with major structural trends and are spatially associated with hydrothermal alteration, indicating their significance in guiding mineralization processes.

### Alteration mapping using ASTER data

#### Spectral ratio indices (SRIs)

**Fig. 9:**
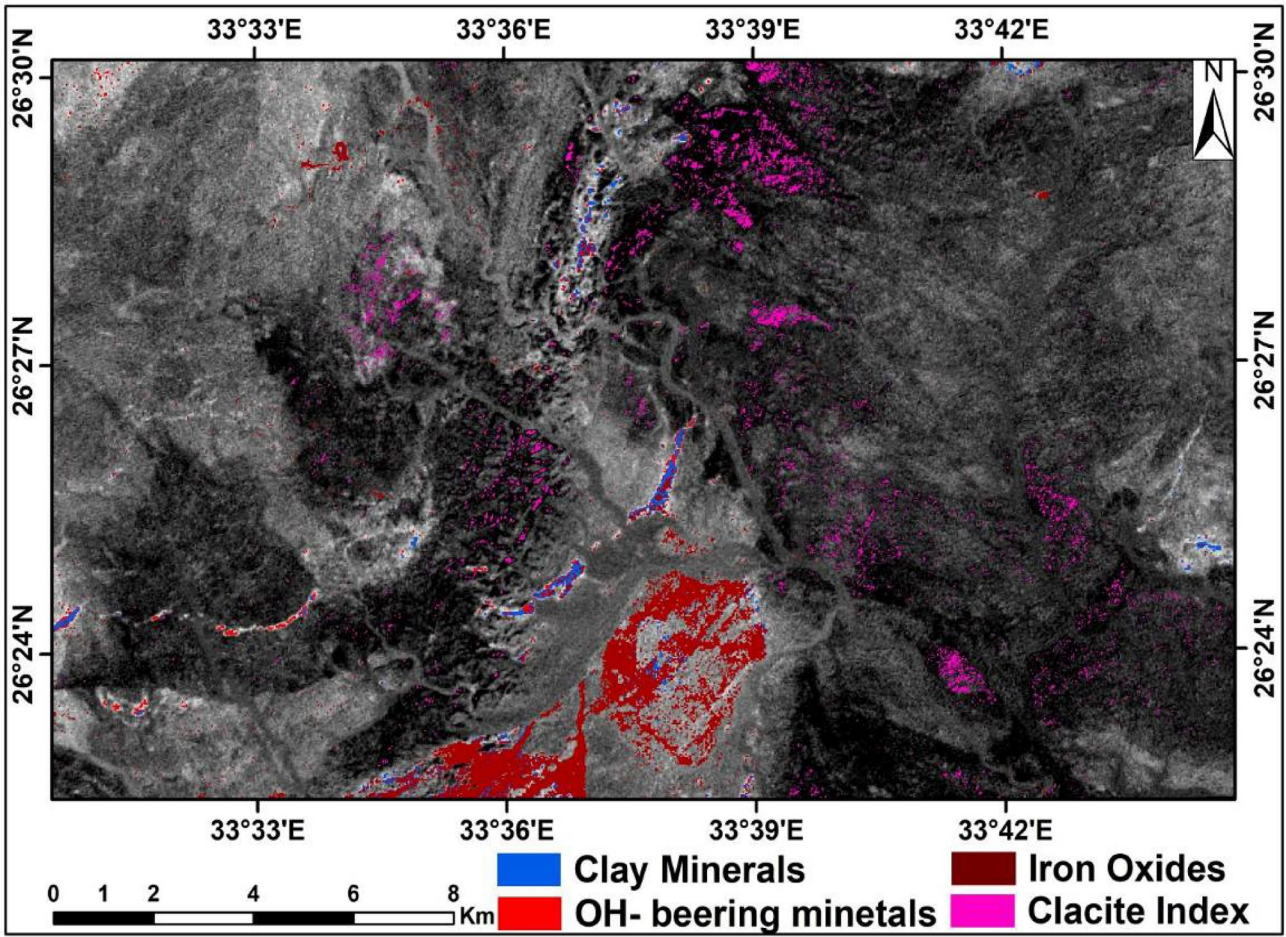
Altered minerals distribution over the study area using the spectral ratio indices method. (By ENVI https://www.l3harrisgeospatial.com/Software-Technology/ENVI).

Several mineralogical spectral ratio indices have been proposed to map alteration zones and associated altered minerals, where altered minerals are present^39,40^. In this study, the OHI, KLI, and CLI indices, in addition to the iron oxide indices of b2/b1, were applied for alteration mapping. The interpretations of such applications reveal that the distributions of OH-bearing, kaolinite, and iron oxide minerals are associated mainly with acidic rocks (Fig. 9). Where OHI is the index of the OH-bearing minerals, KLI is the index of kaolinite minerals, and CLI is the index of calcite minerals.

#### Constrained energy minimization (CEM)

**Fig. 10:**
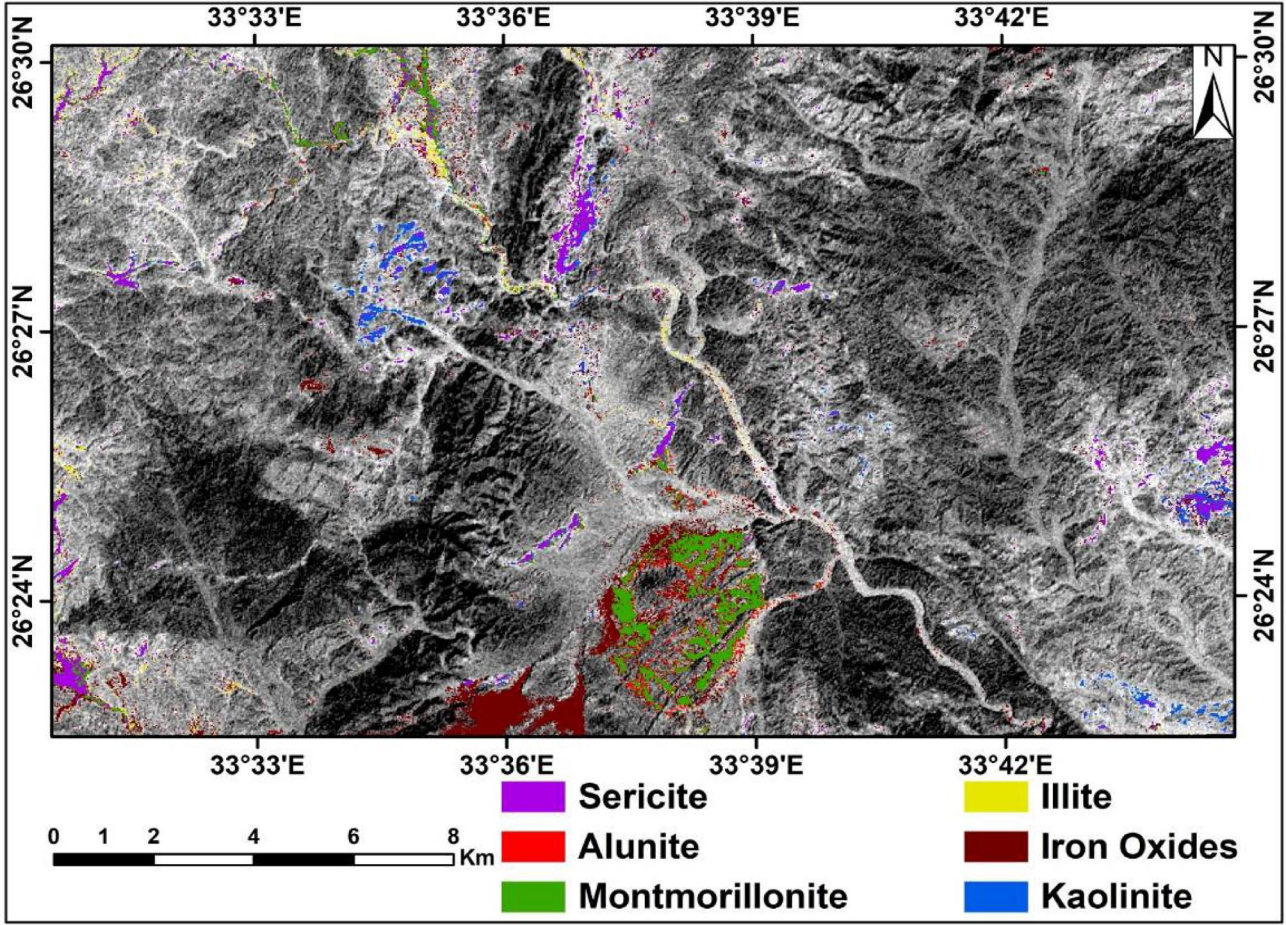
Altered minerals distribution over the study area using the CEM overlying ASTER band 3. (By ENVI https://www.l3harrisgeospatial.com/Software-Technology/ENVI).

The CEM method focuses on the spectral response of specific features while suppressing the responses of other features, treating them as unknown backgrounds^41^. In this study, the CEM method of ASTER VNIR-SWIR surface reflectance data enabled the identification and mapping of the distributions of the most dominant altered minerals iron oxides, montmorillonite, sericite, illite, alunite, and kaolinite (Fig. 10). The altered clay minerals were found to be associated with acidic metavolcanic and Dokhan volcanics. Iron oxides and carbonates are mainly present in the western part, where the intermediate metavolcanics and ultramafic rocks are exposed. Sericite and kaolinite are recorded in a belt trending NE‒SW directly to the north of Gabal Abu Qarahish. In the metavolcanics and the older granites northward of Gabal Semna, there are altered minerals, including kaolinite, montmorillonite, and sericite, associated with the alteration zones.

#### Crosta principal component analysis (CPCA)

This study has harnessed the power of PCA to unravel the intricate spectral signatures of alteration minerals^42^. Drawing from the extensive resources of the US Geological Survey (USGS) spectral library, these endeavors meticulously identified optimal bands that encapsulate the maximum reflectance, and absorption features specific to each alteration mineral. For instance, the spectral behavior of kaolinite is discerned by its pronounced reflectance patterns in bands 4 and 7, juxtaposed with lower reflectance in bands 6 and 9 within the VNIR-SWIR spectral range (Table 5). Such meticulous observations, facilitated by the PCA process using four input bands, culminate in its vivid representation. Similarly, illite, alunite, montmorillonite, and hematite exhibit unique spectral characteristics, all of which are meticulously discerned through the intricate PCA technique.

**Table 5:** Eigenvector statistics of selected ASTER bands 4, 6, 7 and 9 for kaolinite.

Eigenvector	B4↑	B6↓	B7↑	B9↓	Stdev	Eigenvalue	Eigen%
PC 1	0.9624	-0.18021	0.189971	-0.07225	0.080356	0.006967	97.8
PC 2	-0.18756	-0.14708	0.924177	0.298472	0.016923	0.000079	1.1
PC 3	-0.10212	-0.92265	-0.25499	0.270689	0.017982	0.000067	0.9
PC 4	-0.16787	-0.30758	0.211639	-0.91237	0.007529	0.00001	0.2

Kaolinite exhibits significant reflectance characteristics in bands 4 and 7, with notably lower reflectances in bands 6 and 9 within the VNIR-SWIR spectral region. These spectral bands were subjected to principal component analysis (PCA), which yielded four principal components (PCs), as detailed in Table 5. PC2 demonstrated the highest positive loadings from band 7 (0.924177) and band 9 (0.298472), resulting in the representation of kaolinite as blue in the resultant image.

Similarly, illite displays pronounced reflectance in bands 4 and 7, alongside reduced reflectance in bands 6 and 8. The PCA conducted with these bands, as summarized in Table 6, revealed that PC3 exhibited the highest loadings from band 7 (0.966556) and band 6 (0.206267), depicting illite as yellow in the resultant imagery.

**Table 6:** Eigenvector statistics of selected ASTER bands 4, 6, 7 and 8 for illite.

Eigenvector	B4↑	B6↓	B7↑	B8↓	Stdev	Eigenvalue	Eigen%
PC 1	0.982195	0.090829	-0.15934	-0.04065	0.084914	0.007471	97.6
PC 2	0.11263	-0.96656	0.179984	-0.14381	0.012498	0.000098	1.3
PC 3	0.13479	0.206267	0.966556	-0.07109	0.016143	0.000072	0.9
PC 4	-0.06663	0.122327	-0.08935	-0.98621	0.005215	0.000013	0.2

Alunite is characterized by high reflectance in bands 3 and 7 coupled with low reflectance in bands 5 and 9. The PCA results presented in Table 7 indicate that PC3 displayed significant positive loadings from band 7 (0.953996) and band 9 (0.294808), indicating that the alunite was a large sky (red).

**Table 7:** Eigenvector statistics of selected ASTER bands 3, 5, 7 and 9 for alunite.

Eigenvector	B3↑	B5↓	B7↑	B9↓	Stdev	Eigenvalue	Eigen%
PC 1	0.972396	0.126403	-0.10135	0.167918	0.076147	0.006129	94.7
PC 2	0.098645	-0.98439	-0.06548	0.130245	0.019146	0.000277	4.3
PC 3	0.051042	-0.01934	0.953996	0.294808	0.010675	0.000054	0.8
PC 4	0.205209	-0.12096	0.274464	-0.93163	0.013904	0.000013	0.2

Montmorillonite similarly shows high reflectance in bands 3 and 7, with low reflectance in bands 6 and 9. PCA outlined in Table 8, revealed that PC3 exhibited high positive loadings from band 7 (0.979289) and band 9 (0.200399), resulting in the representation of montmorillonite as green.

**Table 8:** Eigenvector statistics of selected ASTER bands 3, 6, 7 and 9 for montmorillonite.

Eigenvector	B3↑	B6↓	B7↑	B9↓	Stdev	Eigenvalue	Eigen%
PC 1	0.972406	0.141543	-0.06403	0.174045	0.075245	0.005983	94.6
PC 2	0.114281	-0.98187	-0.03487	0.147178	0.019331	0.000263	4.2
PC 3	0.028821	-0.00139	0.979289	0.200399	0.009408	0.000066	1
PC 4	-0.20133	0.1261	-0.18888	0.952831	0.014152	0.000012	0.2

Hematite has high reflectance in bands 2 and 4 and low reflectance in bands 3 and 9. The PCA of these bands, detailed in Table 9, indicated that PC2 had high positive loadings from band 3 (0.918021) and band 4 (0.395329), which resulted in hematite appearing brown in the final image.

**Table 9:** Eigenvector statistics of selected ASTER bands 2, 3, 4 and 9 for iron oxides.

Eigenvector	B2↑	B3↓	B4↑	B9↓	Stdev	Eigenvalue	Eigen%
PC 1	0.963953	0.090142	-0.24649	0.043718	0.083281	0.007461	95.2
PC 2	0.013996	0.918021	0.395329	0.027491	0.018285	0.000318	4.1
PC 3	-0.25766	0.38613	-0.88159	-0.08547	0.023085	0.000038	0.5
PC 4	-0.06487	0.003844	-0.07582	0.995001	0.005696	0.000018	0.2

**Fig. 11:**
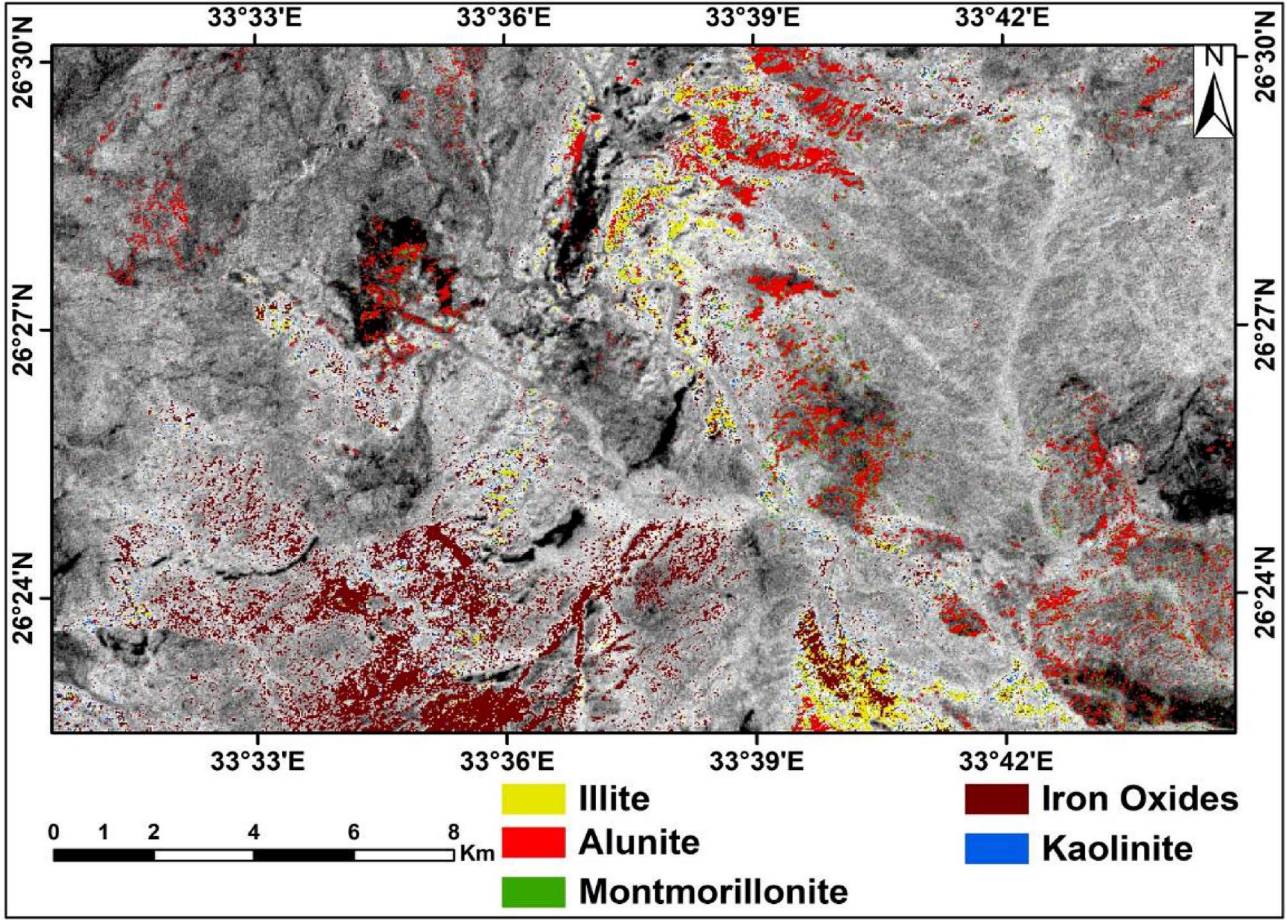
Altered minerals distribution over the study area using the CPCA overlying ASTER band 3. (By ENVI https://www.l3harrisgeospatial.com/Software-Technology/ENVI).

The application of the CPCA method to ASTER data for mapping altered minerals revealed significant spatial coincidence among these minerals. This analysis effectively delineated alteration zones (Fig. 11) that could be used for gold exploration at Gabal Semna and Gabal Abu Karahish, providing critical insights for targeted geological assessments.

### Field verification and mineralogical analysis

The obtained lineament density map was correlated with the provided altered mineral abundance map, which revealed an intimate spatial relation where dense lineaments are confined by the location and distribution of alteration zones. The results of such integration revealed several alteration zones regarded as potentially high-elevation areas for mineral exploration. The known Semna and Abu Qarahish mine areas are delineated areas.

**Fig. 12:**
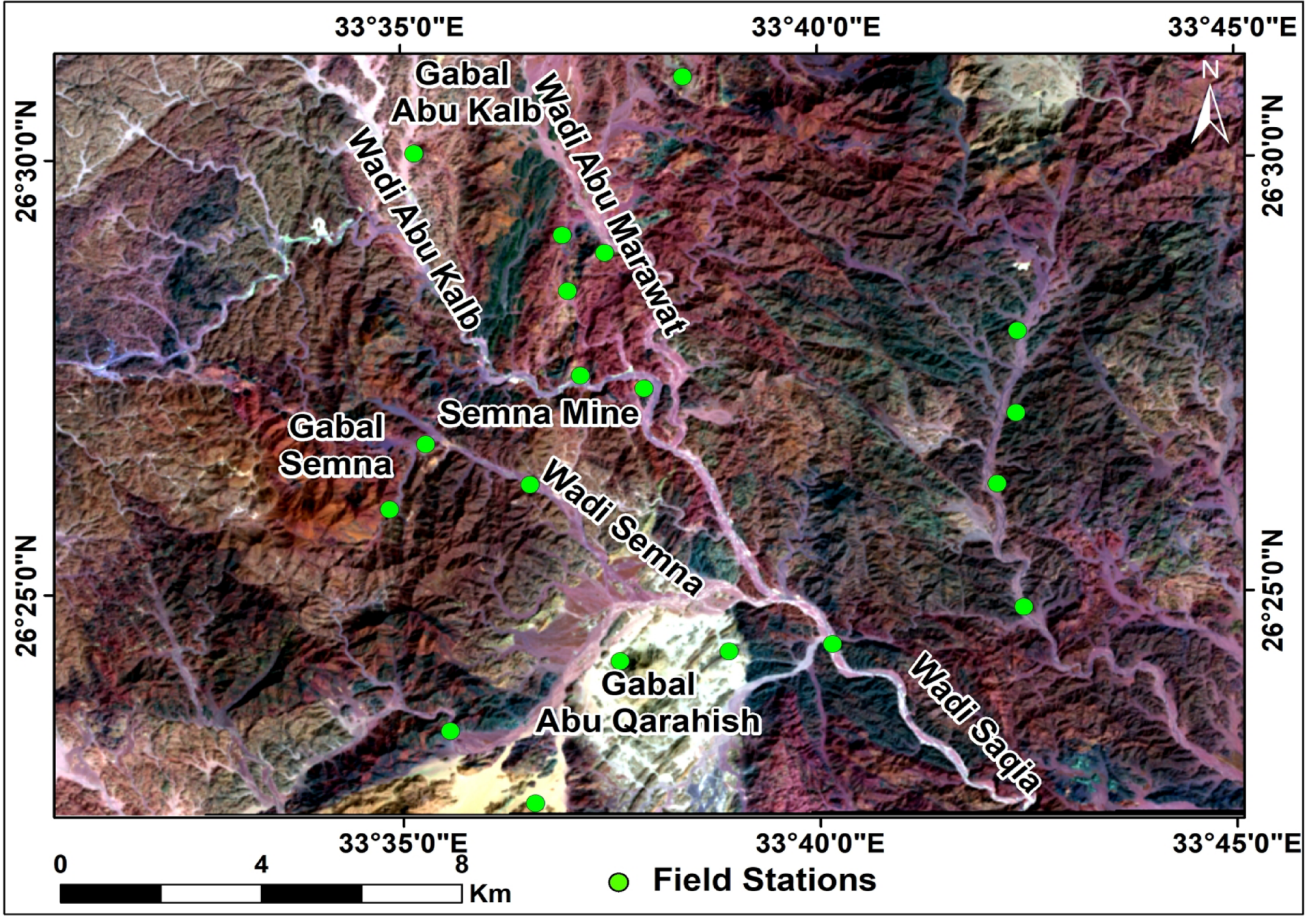
Field station from which samples were collected and observations were recorded. (By ArcGIS v.10.5. https://www.esri.com/en-us/arcgis/products/arcgis-desktop/overview/).

Some field visits have been assumed to verify the results from remote sensing and to locate structurally controlled alteration zones and associated altered minerals. Moreover, representative samples were collected from the quartz veins and alteration zones of various rocks for SEM analysis and gold detection (Fig. 12).

**Fig. 13:**
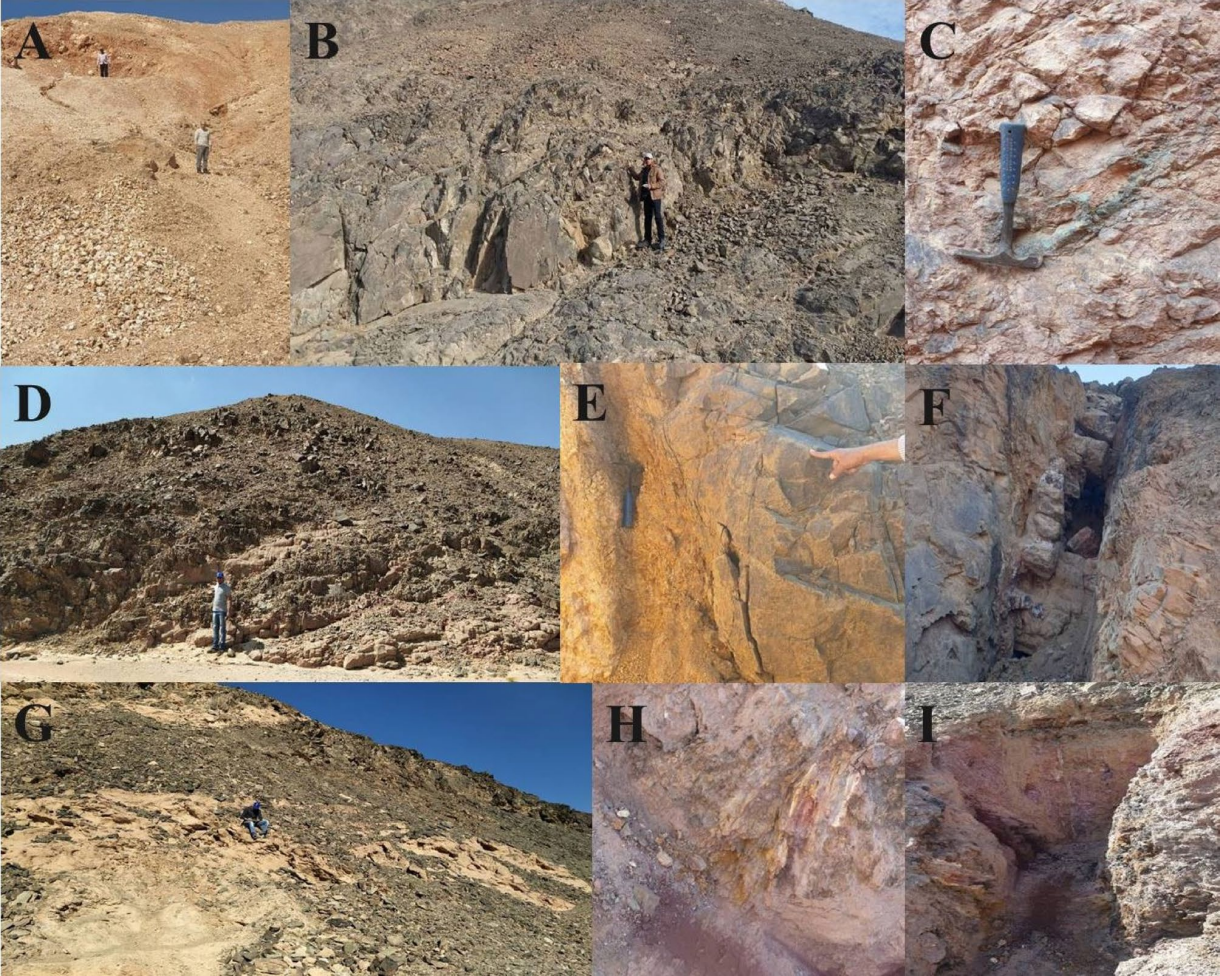
**(A)** Milky and smoky Quartz plug enriched in sulphide and iron oxides near the granitic intrusion of the metavolcanics in the north Gabal Semna area, looking east. **(B)** metavolcanics intruded by metagabbro, in the north Gabal Semna area. **(C)** Alteration zones of metavolcanics in the north Gabal Semna area. **(D)** Younger granites intruding the metavolcanics, in the north Gabal Semna area. **(E) & (F)** Alteration zones of iron carbonate silica (listwaenite ridges), G. Semna area. **(G)** Thrust sheets of serpentinite and talc carbonates over the metavolcanics at G. Abu Karahish area. **(H) & (I)** Alteration zones of (listwaenite ridges), at G. Abu Karahish area.

In the Gabal Abu Karahish area, the metavolcanics are partly overthrusted by serpentinite and talc carbonate Massifs, where several alterations in iron carbonate and silica (listwanite ridges) developed along contacts with the metavolcanics, forming a valid geological environment for gold exploration. The metavolcanics intrude younger granite plutons and several felsite sheets and plugs in the Gabal Abu Qarahish, Gabal Wae’ra and Gabal Kab Amiri areas (Fig. 13). Moreover, the gabbroic and older granitic plutons intrude the acidic metavolcanic and ultramafics rocks in the northern Gabal Semna area. Near the intrusions in the metavolcanics, there are several quartz veins, veinlets, plugs and offshoots of milky and smoky type crosscutting the host rocks of different sizes with varied trends; they are enriched in sulfides and iron oxides, which may enclose gold.

**Fig. 14:**
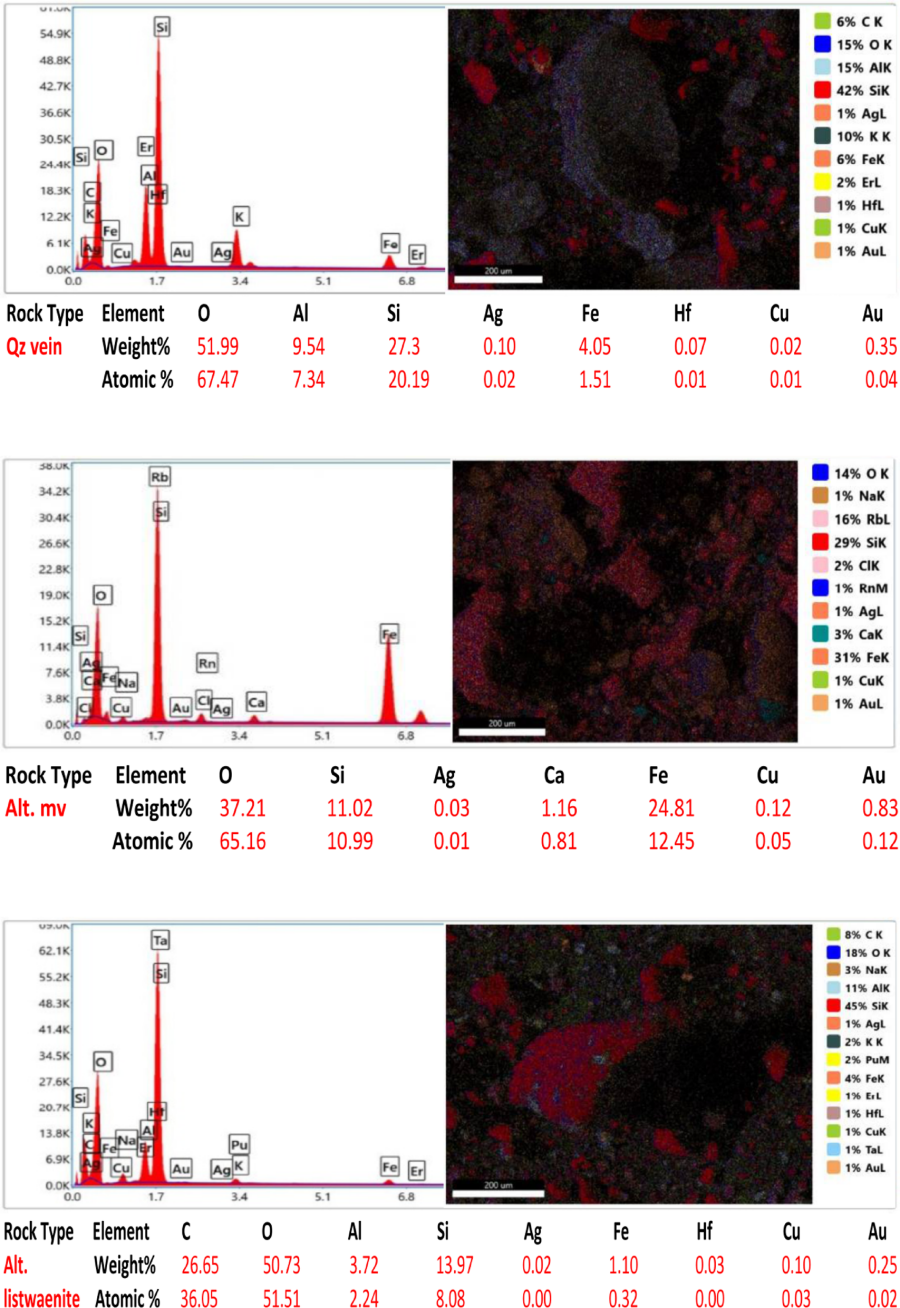

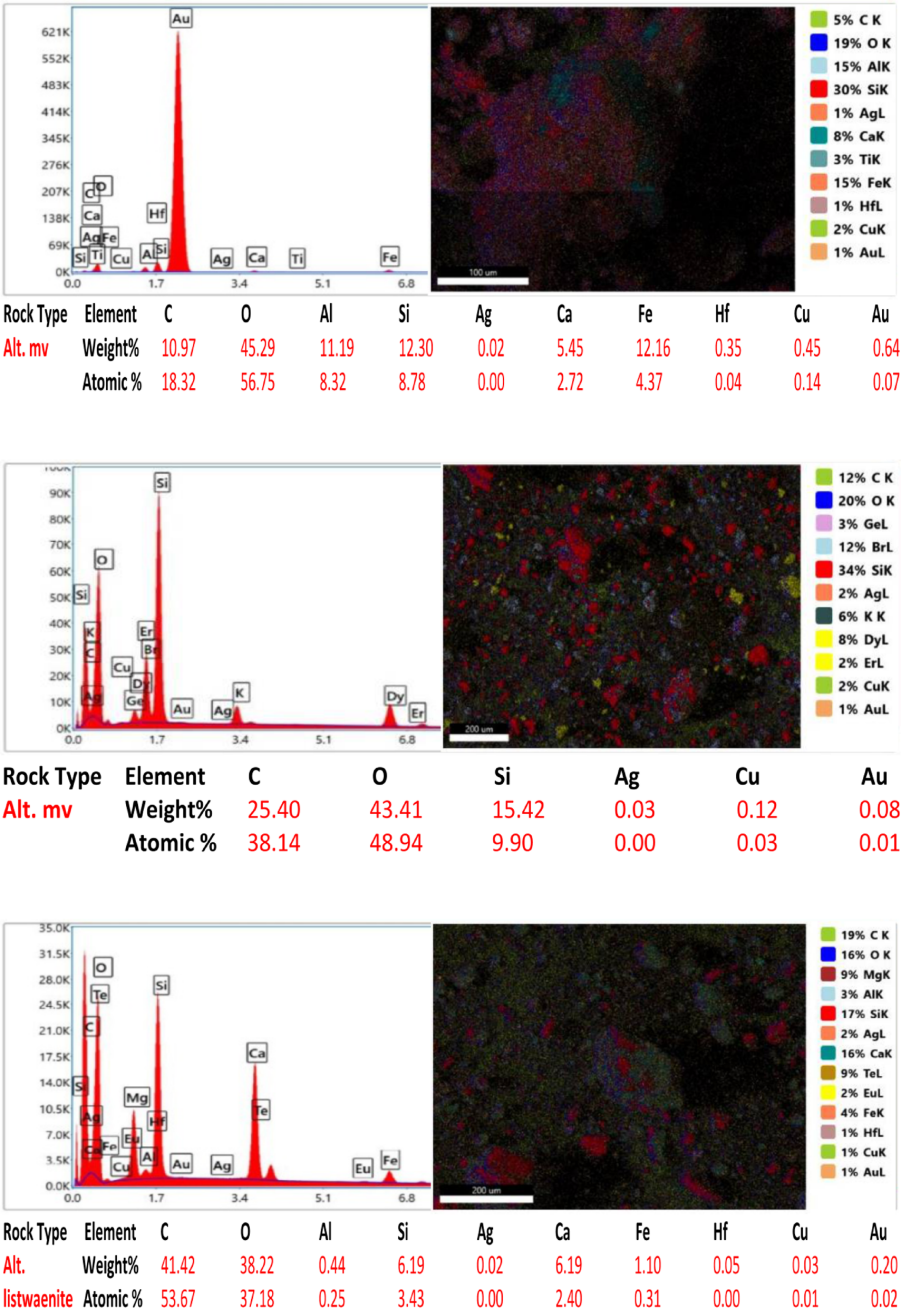

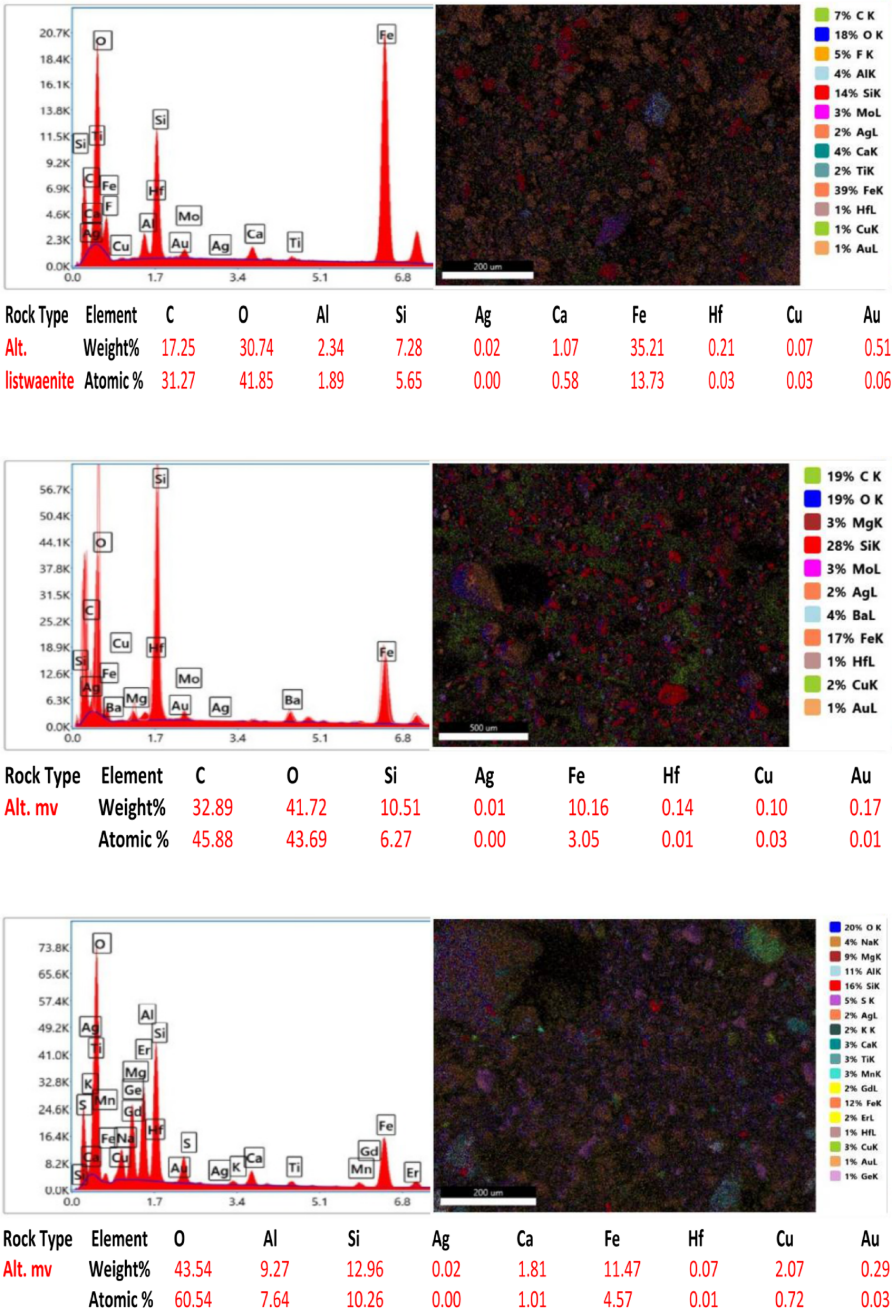

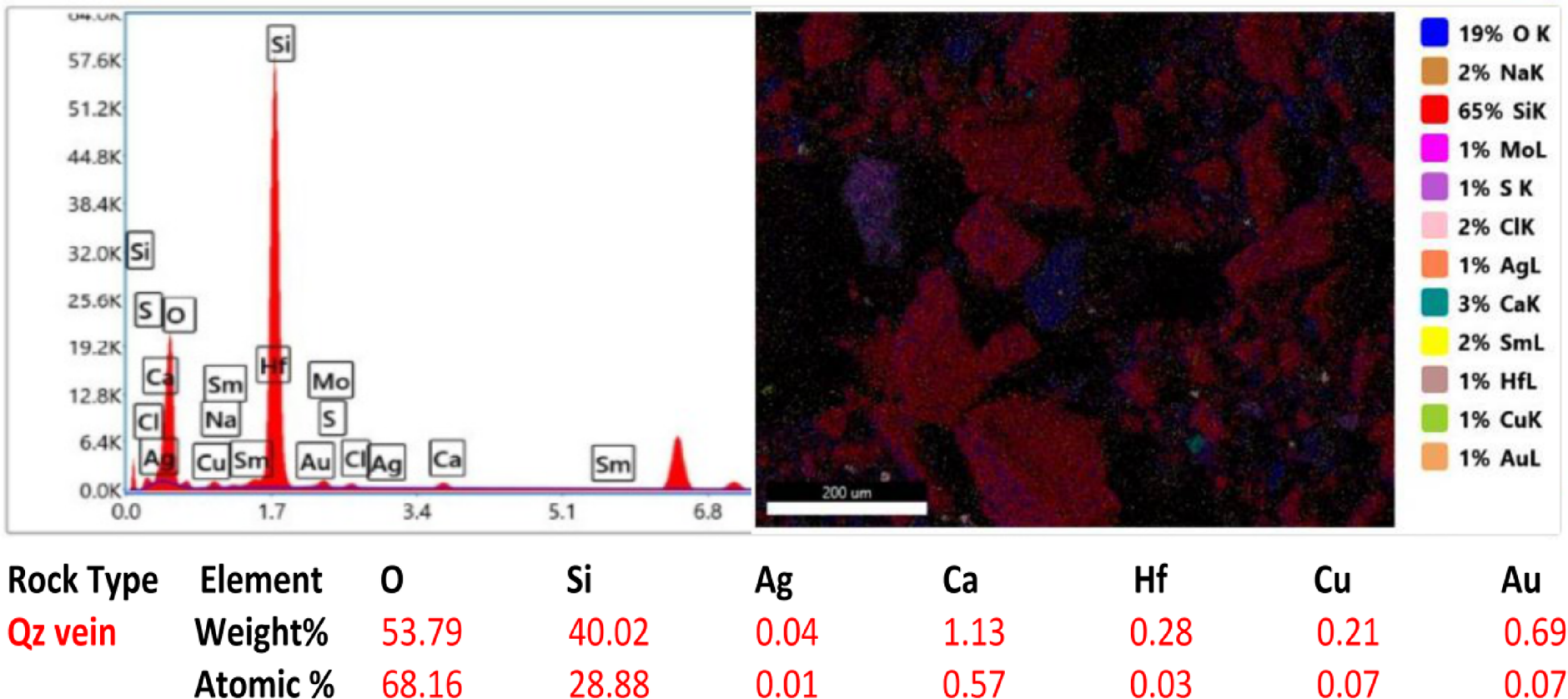
SEM images, sum spectrum and SEM analyses data of collected rock samples.

Ten samples from the altered zones, quartz veins, and country rocks were exposed via electron microscopy (SEM) to detect and assess the gold content and related elements in the study area. All the rock samples contained less than 1% Au in a total bulk of 50 g for each crushed sample. The samples analyzed have gold contents ranging from 0.08 to 0.83% and silver contents ranging from 0.01 to 0.10% (Fig. 14). Sample 2 from the altered metavolcanics contains the highest gold value of 0.83% Au ppm, while Sample 5 from the altered metavolcanics also has a lower Au content of 0.08 ppm. The gold is mainly hosted in pyrite and arsenopyrite crystals and is associated with the following elements at different percentages and randomly related to gold. However, a reverse relationship between gold and Si% is shown in most samples due to the alteration and increase in clay minerals at the expense of silica. The copper (Cu) content ranged from 0.02 to 2.07%, the aluminum (Al) content ranged from 0.44 to 11.19%, the oxygen (O) content ranged from 30.74 to 53.79%, the carbon (C) content ranged from 10.97 to 41.42%, the calcium (Ca) content ranged from 1.07 to 6.19%, the silicon (Si) content ranged from 6.19 to 40.02%, the iron (Fe) content ranged from 1.10 to 35.21%, and the hafnium (Hf) content ranged from 0.03 to 0.28%.

In the alteration zones, a relatively greater percentage of gold is found in the alteration zones than in the host rocks, and more or less than in the quartz veins. In the studied altered zones, silver and copper are frequently connected to gold. Ilmenite, rutile, cobaltite, and barite are occasionally found along with sulfides and iron oxides (the host minerals of gold). The contents of Au, Ag, and their related elements are comparatively high in the altered and quartz vein samples.

## Discussion

Exploring new gold fields in the alteration zones of the areas surrounding the Gabal Abu Karahish and Semna mines is the goal of this study. For this purpose, enhanced mapping of lithological units was necessary for mapping alteration zones as target areas for gold exploration^1,3,4,7,12,24^. In this way, several remote sensing techniques were useful for obtaining Landsat-9 satellite and ASTER data for accurate geological and alteration mapping^2,18,24,25^. Moreover, geological field verification and SEM analysis were performed to support the existence and localization of the alteration zones and their gold potentiality in the study area. The granitic and gabbroic intrusions in the country rocks are mainly associated with mineral-bearing hydrothermal fluids and residual silica that percolate and ascend through rock lineaments and fractures^4,6,7,33,36,41^. The chemical activity and metasomatism of the solutions to the surrounding rocks leave marks of alteration hallows with altered and valued mineral precipitation along the hydrothermal flowing zones^4,14,18,30^. Moreover, sulfide-bearing quartz veins and associated hydrothermal breccia bodies exhibit a variety of textures, including sheared, badinage, and recrystallized quartz, as well as open-space filling and micro breccia within the alteration zones^9,12,13,20^. These textures indicate a complex crack-seal mechanism that highlights the interaction between mineral deposition and a significant N–S-trending shear zone. This relationship is a result of a late brittle–ductile deformation event that impacted the area approximately 550 million years ago. These alteration zones, characterized by variable textures, are promising targets for gold exploration^1,8,11,23,38^. The automatic extraction of the lineaments was also implemented in the localization of the alteration zones, where the rock lineaments represent zones of deformation that imply secondary porosity as channels for the migration of hydrothermal solutions that help in forming and developing alteration zones along their paths^28,35,37^.

Moreover, the alteration and lineament data were integrated to delineate the most potential localities for gold exploration, where the special distribution and density of the extracted lineaments are mainly coincident and correlated with the presence of alteration zones and altered minerals in the study area. At a temperature of approximately 250 °C and pressure of 2.3 kbars, shear zones, and associated alteration zones, combined with the upward migration of hydrothermal fluids, led to a pressure drop^13,14,21^. This pressure drop caused the separation of an aqueous phase and a carbonic phase. The resulting alteration assemblage includes quartz, chlorite, pyrite, calcite, and possibly epidote, with pervasive silicification, pyritization, sericitization, and carbonatization processes contributing to hydrothermal alteration. Additionally, sets of quartz veins and altered shears are significant indicators of hydrothermal alteration. In the El Sid gold mine area, following the emplacement of granite, hydrothermal fluids interacted with sheared serpentinite. These interactions led to metal leaching and re-deposition in adjacent wall rocks and faults through cyclic processes. Alteration zones in the study area were mapped using various remote sensing approaches, including spectral ratio indices (SRIs), constrained energy minimization (CEM), and Crosta principal component analysis (CPCA). The results from fieldwork and SEM analysis corroborated these remote sensing findings. Specifically, the alteration zones identified using ASTER data were verified through field observations, confirming their locations and association with gold mineralization.

## Conclusions

1. The VNIR and SWIR bands of ASTER data are highly effective in mapping hydrothermal alteration zones and detecting altered minerals. These capabilities make ASTER particularly valuable for identifying restricted and localized alteration features associated with mineralization.
2. Specific ASTER band ratios, such as (b7 + b9)/b8, are effective in detecting carbonate, chlorite, and epidote minerals, while (b5 + b7)/b6 highlights sericite and clay minerals. The 4/5 band ratio is especially useful for detailed mapping of alteration zones.
3. Remote sensing techniques including SAM, SFF, CEM, and MTMF efficiently map the spatial distribution of spectral endmembers using ASTER data, enhancing the delineation of hydrothermal alteration zones.
4. Gold occurrences are associated with two primary geological settings: altered metavolcanic rocks and crosscutting quartz veins within country rocks. These alterations include sericitization, chloritization, silicification, and carbonatization, commonly linked to fracture fillings.
5. Extensive alteration zones in ultramafic and metavolcanic rocks, particularly in the Gabal Abu Karahish and Gabal Semna areas, are identified as promising targets for gold mineralization. This is supported by SEM analysis, which revealed gold concentrations of approximately 1% in selected samples.
6. ASTER data proved to be more effective than Landsat-9 in mapping alteration minerals, largely due to the greater spectral richness of ASTER’s six SWIR bands, which provide superior discrimination of hydrothermally altered minerals. However, a limitation of the current methodology is that it primarily relies on remote sensing techniques, which may miss small-scale alterations or those hidden beneath thick surface cover.
7. A methodological limitation of this study is the lack of validation for automatically extracted lineaments. Due to logistical constraints during the field campaign, which prioritized the investigation of hydrothermal alteration zones, field validation or manual extraction was not conducted.
8. Future work should focus on integrating remote sensing results with detailed geophysical surveys, and geochemical investigations to further refine alteration zone delineation and improve exploration targeting. These additional efforts will enhance the accuracy of identifying potential gold mineralization and fully assess the mineral potential of the identified alteration zones.

## Data Availability

The data is available on request from the corresponding author.
